# Apolipoprotein A1-Unique Peptide as a Diagnostic Biomarker for Acute Ischemic Stroke

**DOI:** 10.3390/ijms17040458

**Published:** 2016-03-28

**Authors:** Xu Zhao, Yue Yu, Wenlong Xu, Lei Dong, Yuan Wang, Bing Gao, Guangyu Li, Wentao Zhang

**Affiliations:** 1 School of Bioscience and Bioengineering, South China University of Technology, Guangzhou 510006, China; z.x41@mail.scut.edu.cn (X.Z.); yueyu9023@gmail.com (Y.Y.); yuanyuan03_03@163.com (Y.W.); binggao@scut.edu.cn (B.G.); 2 Department of Neurosurgery, First Affiliated Hospital of China Medical University, Shenyang 110001, China; xwlcmu06@sina.com (W.X.); np-2813@163.com (L.D.)

**Keywords:** apolipoprotein A1 unique peptide (APOA1-UP), acute ischemic stroke (AIS), biomarker, high-density lipoprotein cholesterol (HDL-C), multiple reaction monitoring (MRM), labeled reference peptide (LRP)

## Abstract

Clinically-informative biomarkers of ischemic stroke are needed for rapid diagnosis and timely treatment. In the present study, APOA1 unique peptide (APOA1-UP), a novel peptide biomarker, was identified and quantified by multiple reaction monitoring (MRM) using labeled reference peptide (LRP). Serum samples of 94 patients in the ischemic stroke group and 37 patients in the non-stroke group were analyzed for the levels of total APOA1-UP, low density lipoprotein cholesterol (LDL-C), triglycerides (TG), high-density lipoprotein cholesterol (HDL-C), and total cholesterol (TC). Median ratio of total APOA1-UP/LRP was 2.14 (interquartile range, 0.40) in the non-stroke group and 1.32 (0.44) in the ischemic stroke group (*p* < 0.0001). The serum level of total APOA1-UP was independently correlated with the presence of ischemic stroke by multivariate logistic regression analysis (*p* < 0.0001). From the receiver operating characteristic (ROC) curve, the area under the curve (AUC) was 0.9750 and the optimal cutoff value of the serum APOA1-UP level was 1.80, which yielded a sensitivity of 90.63% and a specificity of 97.14%. The diagnostic efficiency of HDL-C was lower, with an AUC of 0.7488. Therefore, the serum level of APOA1-UP is a diagnostic biomarker candidate for ischemic stroke in the early stage.

## 1. Introduction

Apolipoprotein A1 (APOA1) is the major protein component of high-density lipoprotein (HDL) complexes and plays an important role in the transportation of cholesterol from peripheral tissues to the liver for excretion or reutilization. In several cross-sectional studies, higher serum levels of APOA1 and HDL-C were found in healthy subjects and considered as protective factors, whereas lower levels of APOA1 and HDL-C were associated with higher risks of the acute onset of ischemic stroke [1,2]. Other blood lipids, including low-density lipoprotein cholesterol (LDL-C), triglycerides (TG), and total cholesterol (TC), were related to higher prevalence of ischemic and/or hemorrhagic stroke [3,4]. However, Pikula *et al*. [5] suggested that LDL-C, TG, or TC was not associated with the risk of incidental ischemic stroke, but HDL-C was an important risk factor for ischemic stroke.

APOA1 is more sensitive than HDL-C in the prediction of acute myocardial infarction because its serum level is independent of fasting status and, thus, more feasible in emergency settings [6,7,8]. The APOA1 protein concentration is measured by the enzyme-linked immunosorbent assay (ELISA), an antibody-based clinical assay that is usually expensive and time-consuming. Mass spectrometry is an alternative approach to the development of a protein-specific diagnostic test [9,10]. A potential advantage of mass spectrometry is its ability recognize all possible variations of a target protein. For example, APOA1 protein concentration in serum changes as the new protein is synthesized and the old protein degraded. Proteins are broken down into fragments before they are excreted or recycled [11]. Both integrate and fragmental proteins can be digested into peptides and those peptides, with unique sequences, can be detected and quantified by multiple reaction monitoring (MRM) using triple-quadrupole mass spectrometry. In 2015, Lian *et al*. [12] developed a labeled reference peptide (LRP) method that used a single labeled peptide as a reference standard for all targeted peptides and measured peptides based on their peak areas for detected MRM transitions. Therefore, unique peptides from all possible variants of APOA1 protein in the serum can be identified and quantified by MRM-LRP as a potential clinical laboratory test.

In the present study, patients without stroke were included as the control group instead of healthy subjects to evaluate the clinical diagnostic efficiency. The concentration of total APOA1-UP in the serum was measured by MRM-LRP. First, 1 ng of the reference peptide (APGLTQALNTK) with ^13^C and ^15^N labeling at the C-terminal lysine were added to each injection and the peak area of LRP was measured in each experiment to show the reliability and reproducibility of mass spectrometry analysis. Then, three unique peptides (DYVSQFEGSALGK, AHVDALR, and THLAPYSDELR) were measured to cover integral and all possible fragmental APOA1 proteins. The detection of each unique peptide by MRM is independent and, thus, the mean value of the peak areas of three unique peptides was employed to represent total APOA1-UP in each patient. Finally, the serum level of total APOA1-UP is defined as the peak area ratio of total APOA1-UP to its corresponding LRP (APOA1-UP/LRP). Blood lipid profiles were also detected in both the ischemic stroke group and the control group, and initial computerized tomography (CT) scan was performed as the standard diagnostic test for acute ischemic stroke.

## 2. Results

### 2.1. Demographic Characteristics and Mass Spectrometry Measurements of the Study Population

A total of 144 patients were consecutively admitted and 13 patients with subarachnoid or intracranial hemorrhage, cerebral venous thrombosis, brain injury, transient ischemic attack, or multiple concurrent neurological disorders were excluded. Ninety four patients with acute ischemic stroke were enrolled in this study as the stroke group and 37 patients with peripheral neuropathy, glioblastoma, ependymoma, or meningioma were included as the control group or non-stroke group. The mean age was 57.65 ± 15.14 years and 60.31% were men in the cohort. Patients in both groups were similar in terms of gender, but patients with ischemic stroke were older than patients in the control group. The prevalence of hypertension and previous ischemic heart diseases (IHD) was significantly higher in the ischemic stroke group.

Since the distribution of values for APOA1-UP, LDL-C, TG, HDL-C, and TC were skewed toward the lower end, the median and interquartile ranges were used to describe the variability within samples. The median serum APOA1-UP/LRP was 1.49 (interquartile range (IQR), 0.77) in total samples, 2.14 (IQR, 0.40) in the control group, and 1.32 (IQR, 0.44) in the stroke group, with a *p* value of <0.0001. The serum level of HDL-C was lower in the ischemic stroke group with a value of 1.03 (IQR, 0.54) (mmol/L), close to the low end of the normal range (0.91–1.92) (mmol/L). The median levels of LDL-C, TG, and TC were not statistically significantly different between the stroke group and the non-stroke group (Table 1).

### 2.2. Stratification of the Study Population Across Low, Medium, and High Levels of Serum APOA1-UP

Table 2 presents the number of patients with acute ischemic stroke across three levels of serum APOA1-UP, stratified by age, diabetes mellitus (DM), hypertension, and previous ischemic heart diseases (IHD). Similar probability of ischemic stroke was found in men and women across all the three categories of APOA1-UP serum level. In all of the age strata, the probabilities of acute ischemic stroke changed in a similar trend as the APOA1-UP level increased, which suggest that age was not a confounding factor for the effect of APOA1-UP on ischemic stroke. In patients with DM, or hypertension, or previous IHD, the APOA1-UP level was not associated with the presence of ischemic stroke, which suggested that those diseases were confounding factors.

### 2.3. Inverse Correlation between Serum APOA1-UP Level and the Presence of Ischemic Stroke

Multivariate logistic regression analysis here demonstrated a significant inverse relation between serum APOA1-UP level and the presence of ischemic stroke (*p* < 0.0001), adjusting for age, DM, hypertension, and previous IHD. The odds ratio (OR) of hypertension was 9.39, which suggested that the risk of ischemic stroke was 9.39-fold in patients with hypertension (Table 3).

### 2.4. Evaluation of APOA1-UP as a Diagnostic Biomarker for Acute Ischemic Stroke

**Figure 1:**
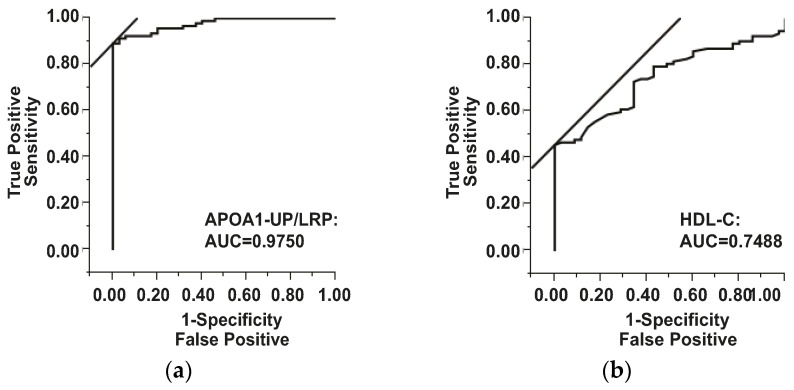
Serum APOA1-UP level and HDL-C level as diagnostic biomarkers for ischemic stroke. Receiver operator characteristic (ROC) curve demonstrating sensitivity as a function of 1-specificity to confirm the diagnosis of ischemic stroke by APOA1-UP/LRP ratio (**a**) or HDL-C level (**b**). APOA1-UP, apolipoprotein A1 unique peptide; LRP, labeled reference peptide; HDL-C, high-density lipoprotein cholesterol; AUC, area under the curve.

Based on the receiver operator characteristic (ROC) curve, the optimal cutoff value of APOA1-UP/LRP ratio as a biomarker for the presence of acute ischemic stroke was projected to be 1.8031 and the area under the curve (AUC) was 0.9750, which yields a sensitivity of 90.63% and a specificity of 97.14% (Figure 1a). Compared to the AUC of HDL-C (0.7488), APOA1-UP has greater discriminatory ability. The cutoff value of HDL-C was 0.9400 (mmol/L), with a sensitivity of 45.83% and a specificity of 97.14% (Figure 1b).

The odds ratio of serum APOA1-UP/LRP ratio (188.13) and its 95% CI (38.04, 930.43) are shown in Table 4. Thus, a serum APOA1-UP level lower than 1.8301, or a positive APOA1-UP test result, was independently related to an increase of 188.13-fold in the probability of ischemic stroke, with a 95% CI of 38.04–930.43. Table 4 also presents the OR of serum HDL-C test (31.68) and its 95% CI (4.17, 240.70). For all the results of initial CT scans, positive findings of ischemic stroke were in 86 out of 94 patients in the stroke group and false negative CT results were found in eight patients with ischemic stroke. Therefore, the sensitivity and specificity of initial CT scan were 91.49% (86/94) and 82.22% (37/45), respectively. For APOA1-UP test, the sensitivity (90.63%) was similar and the specificity (97.14%) was higher, compared with initial CT scan.

## 3. Discussion

In the present study, we determined an inverse association between serum APOA1-UP level and the acute onset of ischemic stroke and found that a decrease in the serum APOA1-UP/LRP ratio was related to an increase in the positive rate of acute ischemic stroke, adjusting for age, DM, hypertension, and previous IHD. The cutoff value of serum APOA1-UP/LRP ratio as a diagnostic biomarker was 1.8031, with a sensitivity of 90.63% and a specificity of 97.14%. The OR of APOA1-UP test was 188.13 and its 95% CI was 38.04–930.43.

### 3.1. Serum APOA1-UP and Initial CT Scans in the Diagnosis of Acute Ischemic Stroke

In this study, initial CT scans at admission were negative for eight patients with acute ischemic stroke (Table 4). Figure S1 shows the means and standard deviations of APOA1-UP/LRP ratios in hyperacute phase (0–12 h), acute phase (>12–24 h), and subacute phase (>24–72 h) of ischemic stroke. The serum level of APOA1-UP was stable during the first 72 h of acute onset of ischemic stroke (*p* = 0.2156). With APOA1-UP test, a quick and convenient diagnosis can be made at the early stage of acute ischemic stroke, even before the detection of infarction by CT, and appropriate treatment can be given as early as possible.

### 3.2. Serum Unique Peptides as Diagnostic Biomarkers for Acute Ischemic Stroke

Blood biomarkers like glial fibrillary acidic protein (GFAP) have been reported as a reliable diagnostic test for hemorrhagic stroke [13]. By MRM-LRP, unique peptides of GFAP, as well as APOA1, can be detected at the same time, and a combination of low APOA1-UP level and normal level of GFAP-UP would suggest a diagnosis of ischemic stroke, whereas a combination of normal APOA1-UP levels and a high level of GFAP-UP would indicate a diagnosis of hemorrhagic stroke.

Such a quick differential diagnosis is important in the treatment of patients with acute ischemic stroke. Computerized tomography (CT) is often normal in the early stage of ischemic stroke or in patients with mild symptoms. MRI is not always feasible, especially for restless patients. Therefore, a rapid blood test to confirm a clinical diagnosis of ischemic stroke is very helpful [14,15,16,17]. With an APOA1-UP test, a quick and convenient diagnosis can be made within the first 12 h of an acute onset of ischemic stroke, even before the detection of infarction by CT, and appropriate treatment can be given as early as possible.

### 3.3. Measurement of Serum Unique Peptides by MRM

In clinical laboratory tests, integral proteins in the human serum are measured by enzyme-linked immunosorbent assay (ELISA), due to its high sensitivity and throughput. However, the availability of highly-specific antibodies for protein biomarker candidates is limited. MRM mass spectrometry has emerged as an alternative to ELISA in biomarker qualification and quantitation [18]. MRM assays identify and measure sequence-specific peptides, or unique peptides, usually with an isotope-labelled unique peptide or reference peptide as a control [9,18,19]. Advantages of unique peptides as stroke biomarkers are as follows: first, they represent both the integral and fragmental endogenous protein pools in the blood; second, peptides and small fragments of proteins move freely across the blood brain barrier (BBB) and can be detected in the serum [20,21]; finally, MRM assays allow measurement of multiple unique peptides at the same time with a high sensitivity. Thus, a panel of unique peptides can be used as a stroke diagnostic test to increase the sensitivity and specificity.

### 3.4. Dyslipidemia and Acute Ischemic Stroke

Dyslipidemia is a well-recognized risk factor of stroke [3]. The serum levels of TC, TG, and LDL-C were higher in patients with ischemic stroke as compared to healthy controls, and the HDL-C level was significantly lower in the stroke group. However, Pikula et.al reported that low HDL-C and a high TC/HDL-C ratio, not LDL-C or TG, were associated with the risk of incident ischemic stroke in a Framingham community cohort study [5]. In 2009, Cuadrado-Godia *et al*. [22] reported that lower TC and LDL were associated with worse prognosis in men. In this study, we found that serum HDL-C level was associated with the acute onset of ischemic stroke, but LDL-C, TC, or TG was not. The serum APOA1-UP level was consistent with the lipid profile findings, which suggested that APOA1-UP can serve as a rapid blood test in cases of emergency.

## 4. Materials and Methods

### 4.1. Materials

Deionized water was produced by a Millipore Advantage A10 system (Millipore, Bedford, MA, USA). Chloral hydrate was ordered from KeLong Chemical (Chengdu, China), 90% ethanol (HPLC grade) from Thermo Fisher Scientific (Rockford, IL, USA), sequencing-grade trypsin from Promega (Madison, WI, USA), ammonium formate from Thermo Fisher Scientific (Rockford, IL, USA), and HPLC-grade acetonitrile (CH_3_CN) from Thermo Fisher Scientific (Rockford, IL, USA). Isotopically-labeled peptide with 99.8% purity: APGLTQALNTK (13C6, 15N2), and unlabeled peptide with the same sequence with 99.0% purity: APGLTQALNTK, were synthesized by GenScript (Nanjing, China).

### 4.2. Study Population

A total of 144 patients were consecutively admitted to the department of neurology at the first hospital of China Medical University from October 2014 to December 2014, in Shenyang, China. All subjects gave their informed consent prior to their inclusion in the study. This study was conducted in accordance with the 1964 Declaration of Helsinki, and the protocol was approved by The Ethics Committee for Medical Research at the First Affiliated Hospital of China Medical University (Shenyang, China). Ninety four patients with ischemic stroke within the first 72 h of acute onset were included as the stroke group, and 37 patients with peripheral neuropathy, glioblastoma, ependymoma, or meningioma were enrolled as the non-stroke group. Demographic characteristics and history of risk factors, including gender, age, diabetes mellitus (DM), hypertension, and previous ischemic heart diseases (IHD), were obtained. Routine blood tests, biochemical tests, and brain CT scans were performed at admission.

### 4.3. Laboratory Tests

Venous blood was collected from all participants within one hour after admission in vacutainer tubes and quickly centrifuged to avoid glycolysis. The serum levels of low-density lipoprotein cholesterol (LDL-C), triglycerides (TG), high-density lipoprotein cholesterol (HDL-C), and total serum cholesterol (TC) were measured by the standard enzymatic colorimetric method using a biochemical analyzer (Roche 7600, Indianapolis, IN, USA). For mass spectrometry analysis, samples containing 40 μg protein were digested and prepared as previously described [12]. Completely digested samples were lyophilized in a speedvac concentrator and then re-suspended in 0.1% formic acid. A total of 1.0 ng/μL APGLTQALNTK* was added to each sample before liquid chromatography/mass spectrometry (LC–MS/MS) analysis.

### 4.4. Selection of Unique Peptides, Reference Peptides, and Q1/Q3 Transition

Three unique peptides for apolipoprotein A1 (DYVSQFEGSALGK, AHVDALR and THLAPYSDELR) with strong MRM transition signals, suitable elution time, and low variability in peak area were selected. Each unique peptide was verified by three MS1/MS2 transition ion pairs. Multiple injections of the digests were performed to measure relative intensities of target ion pair transition signals that had been observed in ion trap MS/MS spectra from the database. Transition signals between different runs were repeated to eliminate the false positive rates of proteins. Collision energy and declustering potential were derived from the empirical equation recommended by Skyline [23]. The parameters of transition ion pairs for apolioprotein A1 unique peptides were provided in supplementary Table 1. Fragment ion-specific tuned Collision Energy (CE) voltages and scheduled retention time were determined for all MRM acquisition methods. All data were acquired with a target cycle time of 1 s, a minimum dwell time of 14 ms, a maximum dwell time of 333 ms (75 maximum concurrent MRMs), and a 300 s MRM detection window for MRM transitions. LC-MRM-MS analysis was performed as previously described [12].

### 4.5. Statistical Analysis

Continuous variables were reported as mean (standard deviation, S.D.) or median (interquartile range, IQR), depending on their normal distribution, whereas categorical variables were expressed as ratios (percentage). Proportions were compared using Fisher’s exact test, and means or medians between groups compared by the Student’s *t* test or the Mann–Whitney *U* test, as appropriate. The independent association between APOA1-UP levels and the presence of ischemic stroke was estimated by a binary logistic regression analysis and the confounding factors were identified with stratification. The Cochran Armitage trend test was used to detect any correlation between serum APOA1-UP level (low, medium, and high) and the probability of ischemic stroke within each stratum. The effect of serum total APOA1-UP on the probability of ischemic stroke was analyzed by multivariate logistic regression, which allows adjustment for confounding factors, such as age, DM, hypertension, and previous IHD. The results are expressed as adjusted odds ratios (OR) and the corresponding 95% confidence intervals (CI). Receiver operating characteristic (ROC) curve and its area under the curve (AUC) were used to evaluate the diagnostic efficiency of the serum APOA1-UP/LRP ratio or HDL-C. The sensitivity, specificity, OR, and 95% CI of serum APOA1-UP levels were compared with those of HDL-C and initial CT scans. JMP 10 (SAS Institute Inc., Cary, NC, USA) software was used for all statistical analysis. *p*-values of <0.05 were considered significant.

## 5. Conclusions

In summary, the serum total APOA1-UP level was inversely correlated with the presence of ischemic stroke, and APOA1-UP can be used as a diagnostic biomarker for acute ischemic stroke at early stage, with a sensitivity of 90.63% and a specificity of 97.14%. In future studies, serum APOA1-UP levels can be measured by MRM coupled with stable isotope dilution (SID) mass spectrometry for absolute quantitation to improve the sensitivity, and the diagnostic potential of total APOA1-UP should be evaluated in patients with other cerebrovascular diseases, such as hemorrhagic stroke, transient ischemic attack (TIA), and cerebral venous thrombosis.

## Figures and Tables

**Table 1:** Demographic characteristics and clinical variables of the study population.

Variables	Total (*n* = 131)	Control (*n* = 37)	Ischemic Stroke (*n* = 94)	*p* ^a^
Age, Mean (SD)	57.65 (15.14)	47.08 (16.09)	61.81 (12.58)	<0.0001
Gender, %				
Male	60.31	62.16	59.57	0.8445
Female	39.70	37.84	40.43	
Diabetes Mellitus, %	12.21	5.41	14.89	0.2338
Hypertension, %	30.53	5.41	40.43	<0.0001
Previous Ischemic heart disease, %	12.21	2.70	15.96	0.0398
LDL-C in mmol/L, Median (IQR)	2.75 (0.82)	2.73 (1.04)	2.77 (0.75)	0.9124
TG in mmol/L, Median (IQR)	1.52 (0.55)	1.45 (0.38)	1.59 (0.63)	0.0619
HDL-C in mmol/L, Median (IQR)	1.06 (0.55)	1.39 (0.59)	1.03 (0.54)	<0.0001
Total-C in mmol/L, Median (IQR)	4.54 (1.03)	4.43 (1.34)	4.56 (0.94)	0.0580
APOA1-UP/LRP, Median (IQR)	1.49 (0.77)	2.14 (0.40)	1.32 (0.44)	<0.0001

^a^ Mann–Whitney *U* test, Student’s *t* test, or Fisher’s exact test was used. Results are expressed as percentages or as mean (standard deviation, SD) and medians (interquartile range, IQR); LDL-C, low-density lipoprotein cholesterol; TG, triglycerate; HDL-C, high-density lipoprotein cholesterol; Total-C, total serum cholesterol; APOA1-UP, apolipoprotein A1 unique peptide.

**Table 2:** Case number of patients across categories of serum APOA1-UP level, stratified by age, diabetes mellitus, hypertension, and previous ischemic heart diseases.

Demographic Characteristics	No. of Patients		Serum APOA1-UP/LRP Ratio						*p* ^a^ for Trend
			Low		Medium		High		
	I.S.	Ctrl.	I.S.	Ctrl.	I.S.	Ctrl.	I.S.	Ctrl.	
Overall	94	37	14	0	76	10	4	27	<0.0001
Gender									
Male	56	23	12	0	42	8	2	15	<0.0001
Female	38	14	2	0	34	2	2	12	<0.0001
Age									
0–29	1	4	0	0	1	0	0	4	0.0253
30–59	44	26	5	0	38	6	1	20	<0.0001
60–89	49	7	9	0	37	4	3	3	0.0216
Diabetes Mellitus									
Yes	14	2	2	0	12	4	0	0	0.4489
No	80	35	12	0	12	8	4	27	<0.0001
Hypertension									
Yes	38	2	4	0	33	1	1	1	0.1297
No	56	35	10	0	43	9	3	26	<0.0001
Previous IHD									
Yes	15	1	3	0	11	1	1	0	0.7418
No	79	36	11	0	65	9	3	27	<0.0001

^a^ Cochran–Armitage trend test was used. Results are expressed as percentages or as means (SD); I.S., ischemic stroke; Ctrl, control; APOA1-UP, apolipoprotein A1 unique peptide; LRP, labeled reference peptide; IHD, ischemic heart diseases. Serum APOA1-UP/LRP level: Low, 0–<1; Medium, 1–<2; High, 2–3.

**Table 3:** Adjusted odds ratio (OR) and CI of confounding factors.

Variables	OR	95% CI	*p*
Age	1.03	0.97, 1.10	0.3376
Diabetes Mellitus	0.46	0.03, 11.74	0.5824
Hypertension	9.39	1.12, 150.04	0.0382
Previous IHD	3.40	0.19, 119.24	0.4184

OR, odds ratio; CI, confidence interval; IHD, ischemic heart diseases.

**Table 4:** Evaluation of APOA1-UP/LRP, HDL-C, and initial CT scan as diagnostic tests for acute ischemic stroke.

Variables	APOA1-UP/LRP	HDL-C	CT ^a^
AUC	0.9750	0.7488	–
Sensitivity	0.9063	0.4583	0.9149 (86/94)
Specificity	0.9714	0.9714	0.8222 (37/45)
Diagnostic Index	1.8777	1.4298	1.7371
Cut-off Value	1.8031	0.9400	–
OR	188.13	31.68	–
95% CI	38.04, 930.43	4.17, 240.70	–
*p* ^b^	<0.0001	<0.0001	<0.0001

^a^ Initial computed tomography taken at admission; ^b^ Fisher’s exact test; OR, odds ratios; CI, confidence interval; APOA1-UP, apolipoprotein A1 unique peptide; LRP, labeled reference peptide; HDL-C, high-density lipoprotein cholesterol.

## Supplementary Materials

Supplementary materials can be found at http://www.mdpi.com/1422-0067/17/4/458/s1.

## Abbreviations

APOA1-UP: apolipoprotein A1 unique peptide
AIS: acute ischemic stroke
HDL-C: high-density lipoprotein cholesterol
LDL-C: low-density lipoprotein cholesterol
TG: triglycerides
TC: total cholesterol
MRM: multiple reaction monitoring
LRP: labeled reference peptide
ROC: Receiver operator characteristic (ROC) curve
AUC: area under the curve
OR: odds ratio
