# Supplementary material for: Apolipoprotein A1-Unique Peptide as a Diagnostic Biomarker for Acute Ischemic Stroke
Source: Int J Mol Sci. 2016 Mar 28;17(4):458. doi: 10.3390/ijms17040458 (PMC4848914; doi:10.3390/ijms17040458)
Supplement: Supplementary file 1 [file ijms-17-00458-s001.pdf]

Supplementary Materials: Apolipoprotein A1-Unique Peptide as a Diagnostic Biomarker for Acute Ischemic Stroke

Xu Zhao, Yue Yu, Wenlong Xu, Lei Dong, Yuan Wang, Bing Gao, Guangyu Li and Wentao Zhang

Table S1. MRM parameters for APOA1-Ups.

Sequences	Parent Ions	Daughter Ions	Collisional Energy (V)	Declustering Potential (V)	Retention Time (min)
DYVSQFEGSALGK1	700.84	1023.51			
DYVSQFEGSALGK2	700.84	808.42	35.7	82.2	13.4
DYVSQFEGSALGK3	700.84	532.31			
THLAPYSDELR1	651.33	1063.54			
THLAPYSDELR2	651.33	950.46	32.9	78.6	5.6
THLAPYSDELR3	651.33	879.42			
AHVDALR1	391.22	573.34			
AHVDALR2	391.22	474.27	18.0	59.6	1.0
AHVDALR3	391.22	359.24			

MRM, multiple reaction monitoring; APOA1-UPs, apolipoprotein A1 unique peptides.
